# Supporting those bereaved by cancer: a service evaluation and investigation of cognitive behavioural mechanisms in the treatment of prolonged grief

**DOI:** 10.1080/20008066.2025.2545144

**Published:** 2025-09-02

**Authors:** Kirsten V. Smith, Graham R. Thew, Sarah C. Carr, Paris R. Congrave, Susie Rudge, Erin H. Thompson

**Affiliations:** a Department of Experimental Psychology, University of Oxford, Oxford, UK; b Oxford Health NHS Foundation Trust, Oxford, UK; c The Loss Foundation [Registered Charity 1147362], London, UK

**Keywords:** Bereavement, grief, prolonged grief disorder, cancer, cognitive behavioural therapy, Duelo, luto, trastorno de duelo prolongado, cáncer, terapia cognitivo-conductual, recuerdo, autocompasión, desconexión social, terapias grupales

## Abstract

**Background:** Individuals bereaved by cancer face significant emotional challenges, often experiencing prolonged grief disorder (PGD), PTSD, depression, and anxiety. Effective interventions are needed to target these mental health problems. This study evaluates the outcomes of the specialist bereavement charity, The Loss Foundation’s therapeutic group intervention designed for individuals grieving a cancer-related loss.

**Methods:** A total of 68 participants, enrolled across five cohorts, received a short-term group intervention targeting cognitive–behavioural factors and self-compassion. Due to recruitment limitations, randomized analyses were underpowered, therefore a broader service evaluation was performed, combining data from 2016 and 2018 cohorts. The primary outcome was PGD symptoms measured by the PG-13, with secondary outcomes examining PTSD, depression, anxiety, and self-compassion. Process measures were memory characteristics, grief appraisals, maladaptive coping strategies, and social disconnection. Data were analysed using linear mixed-effects models.

**Results:** Significant reductions were observed in symptoms of PGD (*d* = 0.65 at 3-month follow-up), PTSD, depression, and anxiety, with improvements in self-compassion (*d* = 0.53). Cognitive–behavioural process measures also showed significant changes, particularly in memory characteristics and negative appraisals, though social disconnection did not significantly change. Exploratory analyses indicated that lower baseline negative appraisals predicted better treatment outcomes. Attrition was minimal after the intervention began, though approximately 25% of participants did not provide follow-up data.

**Conclusions:** The group intervention demonstrated positive effects on grief-related and mental health outcomes, supporting the use of cognitive–behavioural approaches in cancer bereavement. However, further randomized trials with larger samples are needed to confirm these findings and address limitations related to randomization and data completeness.

## HIGHLIGHTS

- This study evaluates a cognitive–behavioural group intervention designed to support individuals grieving a cancer-related loss, with promising improvements in prolonged grief disorder, PTSD, and anxiety.
- Findings demonstrate significant reductions in grief-related symptoms, PTSD, depression and anxiety, alongside increased self-compassion, at both post-treatment and follow-up.
- Changes in loss-related memory characteristics and negative appraisals provide insight into the mechanisms driving treatment success, emphasizing the value of targeted cognitive–behavioural approaches in cancer bereavement.

## Abstract (es)

**Antecedentes:** Las personas en duelo por una pérdida causada por cáncer enfrentan importantes desafíos emocionales, y a menudo presentan trastorno de duelo prolongado (PGD, por sus siglas en inglés), TEPT, depresión y ansiedad. Se requieren intervenciones eficaces para abordar estos problemas de salud mental. Este estudio evalúa los resultados de una intervención grupal terapéutica, desarrollada por la organización benéfica especializada en duelo The Loss Foundation, dirigida a personas que enfrentan un duelo relacionado con el cáncer.

**Métodos:** Un total de 68 participantes, distribuidos en cinco cohortes, recibieron una intervención grupal de corto plazo enfocada en factores cognitivo-conductuales y autocompasión. Debido a limitaciones en el reclutamiento, los análisis aleatorizados carecieron de poder estadístico suficiente, por lo que se realizó una evaluación más amplia del servicio, combinando datos de las cohortes de 2016 y 2018. El resultado principal fue la sintomatología de PGD, medida con el PG-13. Los resultados secundarios incluyeron TEPT, depresión, ansiedad y autocompasión. Las medidas de proceso incluyeron características del recuerdo, evaluaciones del duelo, estrategias de afrontamiento desadaptativas y desconexión social. Los datos se analizaron utilizando modelos lineales de efectos mixtos.

**Resultados:** Se observaron reducciones significativas en los síntomas de PGD (*d* = 0.65 en el seguimiento a 3 meses), TEPT, depresión y ansiedad, junto con mejoras en la autocompasión (*d* = 0.53). Las medidas de proceso cognitivo-conductuales también mostraron cambios significativos, especialmente en las características del recuerdo y en las valoraciones negativas del duelo, aunque la desconexión social no cambió de forma significativa. Los análisis exploratorios indicaron que niveles más bajos de interpretaciones negativas sobre la pérdida al inicio predecían mejores resultados del tratamiento. La tasa de abandono fue mínima después del inicio de la intervención, aunque aproximadamente el 25% de los participantes no entregaron datos de seguimiento.

**Conclusiones:** La intervención grupal mostró efectos positivos en el duelo y en la salud mental, lo que respalda el uso de enfoques cognitivo-conductuales en personas en duelo por cáncer. Sin embargo, se necesitan más ensayos aleatorizados con muestras más grandes para confirmar estos hallazgos y abordar las limitaciones relacionadas con la aleatorización y la completitud de los datos.

## 1. Introduction

Cancer is one of the leading causes of death globally, accounting for approximately 10 million deaths every year (Bray et al., 2024). Losing a loved one to cancer can involve many potentially traumatic moments (i.e. initial diagnosis, terminal diagnosis, physical deterioration, caregiving etc.) (Smith, Rankin, et al., 2020). Given these challenges, individuals bereaved by cancer are particularly vulnerable to post-loss mental health problems such a prolonged grief disorder (PGD), posttraumatic stress disorder (PTSD) (Guldin et al., 2012), and depression (Kim et al., 2014). There is a critical need to understand what therapeutic approaches could be acceptable, beneficial, and efficient for this population and to explore how these interventions work.

Research into the effectiveness of bereavement groups in reducing mental health problems has shown that just attending a group where the discussion of grief is welcomed and encouraged is not sufficient to reduce grief, anxiety, or depression (Näppä & Björkman-Randström, 2020). This suggests that although emotional support is important, it alone does not address the underlying cognitive behavioural processes that contribute to mental health problems after a loss such as loss-related memory characteristics, negative beliefs, maladaptive coping strategies. As a result, there has been a necessary shift towards evidence-based approaches that specifically target these mechanisms which are known to predict and maintain post-loss mental health issues (Smith et al., 2024; Smith & Ehlers, 2021, 2023). These targeted interventions offer the potential for more meaningful and lasting improvements in grief-related outcomes.

The Loss Foundation, a UK-based charity, provides free supportive services to individuals bereaved by cancer. Previous research found their group-based therapeutic support, which incorporates cognitive–behavioural therapy (CBT) significantly improved self-compassion and reduced grief intensity, PTSD, depression, and anxiety from baseline to follow-up in a small sample of individuals bereaved for at least six months. A quasi-experimental waitlist control group showed no change in symptoms over a three-month period (Jerome et al., 2018), providing encouraging initial evidence that this approach may support adaptation after a cancer-related loss.

More recently, Lacasta and Cruzada (2024) conducted a randomized clinical trial to evaluate the effectiveness of Cognitive–Behavioural Group Therapy (CBGT) for complicated grief among relatives of cancer patients. The trial included 249 participants with assessments at baseline, post-treatment, and 6- and 12-month follow-ups. The CBGT group showed significant improvements in complicated grief, depression, anxiety, hopelessness, and general health compared to a psychoeducational control group (Lacasta & Cruzado, 2024). These results emphasize the importance of targeting cognitive–behavioural processes in cancer bereavement and support their use in group settings. However, further research is needed to determine whether these approaches are relevant to newer conceptualizations of severe and enduring grief, such as prolonged grief disorder (PGD) which has now been formally recognized in both the ICD-11 and DSM-5-TR as a distinct mental health condition (Killikelly & Maercker, 2018; Prigerson et al., 2021). It is characterized by persistent and pervasive grief, such as intense longing or preoccupation with the deceased and profound emotional pain, that significantly impairs daily functioning and continues beyond what is considered culturally or socially normative. This diagnostic development reflects a shift from earlier constructs such as ‘complicated grief’ and the provisional DSM-5 diagnosis of persistent complex bereavement disorder (PCBD), marking a growing consensus that severe, enduring grief constitutes a distinct clinical syndrome.

Cognitive–behavioural mechanisms have been implicated in the development and maintenance of PGD (Smith & Ehlers, 2021, 2023). The theory suggests that how individuals think about and remember their loss, as well as their behavioural responses, can drive distressing symptoms and maintain elevated levels of grief. Empirical support for this theory has been demonstrated in treatment studies using Prolonged Grief Disorder Therapy (PGT), previously known as Complicated Grief Therapy. Studies have shown that reductions in grief symptoms and functional impairment are mediated by changes in negative beliefs about the loss and avoidance behaviours (Lechner-Meichsner et al., 2022; Skritskaya et al., 2020). However, little is known about the role of memory integration and social disconnection in psychological treatment for grief.

The Oxford Grief Study identified four key cognitive–behavioural mechanisms that contribute to prolonged grief: loss-memory characteristics, negative appraisals, maladaptive coping strategies, and social disconnection (Smith, 2018; Smith & Ehlers, 2020). These mechanisms align with the cognitive model for PTSD (Ehlers & Clark, 2000) and the cognitive–behavioural model for PGD (Boelen et al., 2006). Loss-related memory characteristics reflect difficulties in integrating the loss into autobiographical memory, often leading to intrusive and distressing memories that are easily triggered. Negative appraisals – such as regret, catastrophic thinking about the future, or viewing grief as overwhelming – are cognitive extremes that can maintain grief symptoms. Maladaptive coping strategies – such as avoidance, proximity-seeking, and rumination – prevent the revision of these beliefs by blocking new learning experiences. Finally, social disconnection, describes feelings of isolation and the belief that expressing grief will be met with rejection, which further limits social support (Wanza et al., 2023) and has been identified as a strong predictor of PGD in next of kin of individuals who died from COVID-19 in intensive care units (Rodriguez-Villar et al., 2024). This study will be the first to use the Oxford Grief Study tools to measure how these specific cognitive–behavioural mechanisms change in response to psychological treatment.

Given this context, our study sought to replicate and further explore the therapeutic group outcomes reported by Jerome et al. (2018). Originally, The Loss Foundation planned a randomized controlled trial (RCT) following the work of Jerome and colleagues. However, due to resource limitations and recruitment difficulties within the required timeframe, the study would have been underpowered for the intended analyses. Therefore, we opted to combine data collected from this study with data from the previous three treatment cohorts reported in Jerome et al. (2018) to conduct a larger-scale service evaluation of five treatment cohorts. While this larger sample did not allow for direct comparisons between waitlist and immediate-start groups, it enabled the use of more sophisticated longitudinal analysis methods to examine within-group changes and the associations between process variables and clinical outcomes. This study aimed to extend previous findings by incorporating two additional therapeutic groups and exploring process-outcome relationships within this intervention.

## 2. Method

### 2.1. Study design

In total, five therapeutic groups for adults bereaved by cancer were delivered. Three took place in 2016, comprised of two cohorts who started immediately following their initial assessment, and one cohort who started following a wait period of three months. Participants were allocated to cohorts based on their availability. In 2018 two further cohorts received treatment, one immediately, and one following a three-month wait period. These participants were randomized to one of the two groups using an online randomization tool (Urbaniak & Plous, 2013).

However, as described above, the numbers treated in the 2018 groups were not sufficient to adequately power the intended RCT analyses so we therefore combined all five cohorts together (*n* = 68) as a broader service evaluation of the therapeutic group protocol. The research received ethical approval from University College London's Research Ethics Committee (project ID: CEHP/2015/530) and The Loss Foundation Research Committee (Project ID No: 180829TLF). The 2018 cohort study was prospectively registered on the ISRCTN registry (ISRCTN56216152).

### 2.2. Participants and procedure

The Loss Foundation is a U.K. charity providing free supportive services to those bereaved by cancer. All participants (*n* = 68) self-referred in response to the charity’s mailing list or social media outreach. Inclusion criteria were age 18 or over, bereavement by cancer a minimum of 6 months prior with no upper limit on time since loss, and no other current psychological treatment. Exclusion criteria were self-reported misuse of drugs or alcohol, or active suicide risk. Ineligible participants were signposted to relevant services.

Participants registered their interest online after reading an information sheet about the study. They were then screened on the telephone by charity employees who explained the nature of the group and checked eligibility criteria. Eligible participants were then sent a link to give their informed consent to participate and complete a set of symptom and process measures. All measures were completed online and collected in accordance with ethical guidelines (Smith et al., 2018). Participants completed measures at baseline, end of treatment (7 or 8 weeks post-baseline) and at 3 months after treatment ended (follow-up). For the waitlist the timing of assessments is as above but there was a 3-month wait between their start of wait data and baseline data collection.

### 2.3. Intervention

The intervention was initially developed by the first and last author as a 6-session weekly group intervention. The core components of the programme focused on psychoeducation of the grieving process (Stroebe & Schut, 1999) and the maintaining processes of Prolonged Grief and PTSD such as a lack of integration of the loss into autobiographical memory (Ehlers & Clark, 2000; Smith et al., 2022) and negative appraisals (Smith & Ehlers, 2023) while fostering the practice of self-compassion (Gilbert, 2009), self-care and emotional wellbeing. At the end of each session, participants were encouraged to plan and put into action a behavioural component of each session’s theme to facilitate consolidation of learning and build confidence in the techniques being taught. Following feedback from group participants in 2016, the groups were extended to 7 sessions to allow for more space for group discussions. The first group session was an opportunity to introduce the group’s aims and ground rules and to allow participants to share their experiences of loss. Each session was 2 h long and consisted of a debrief of the previous session and its associated action plan, an introduction of that week’s materials, the action plan for that week and addressing any questions followed by a mindfulness exercise. Groups were limited to 10–15 participants to allow the capacity for group discussions.

For the 2016 schedule see (Jerome et al., 2018). The 2018 groups were delivered as follows^1^

**Session 1:** Introductions to the group and each other
**Session 2:** Psychoeducation about the grief experience and associated difficulties (e.g. anxiety, low mood,)
**Session 3:** Continued Psychoeducation (e.g. intrusive thoughts and memories) Self-care and routine (e.g. sleep hygiene and activity scheduling)
**Session 4:** Self-compassion – Psychoeducation and developing a perfect nurturer
**Session 5:** Unhelpful cognitions – Private grief and suppression of negative thoughts – strategies for managing different categories of thoughts
**Session 6:** Developing resilience to unhelpful thoughts or memories through exposure
**Session 7:** Reflections and endings (Sharing photos and defining values)

Therapists were all trained by either the first or last author on delivering the groups and were supported to work to protocol via regular supervision. All were qualified psychologists trained in delivering cognitive behavioural therapy. Across the five cohorts there were seven therapists in total with each being facilitated by two therapists. The first author facilitated two groups, the last author facilitated three, and the remaining five therapists facilitated one group each.

### 2.4. Measures

#### 2.4.1. Symptom measures

The primary outcome measure was the *Prolonged Grief-13 Inventory.* The PG-13 *(*Prigerson & Maciejewski, 2008*)* was used to measure symptoms of separation-related distress alongside cognitive and behavioural disturbances following bereavement. We employed a revised version that was adapted to include an additional 6 items mapping the Persistent Complex Bereavement Disorder conceptualization that was added to the DSM conditions for further study section. We selected the ten items best aligned with the criteria outlined in the DSM-5-TR for PGD (American Psychiatric Association, 2022) which we refer to here as the adapted PG-13. A probable diagnosis of PGD has been defined as a score of 30 or more on these items (Prigerson et al., 2021). Scores ranged from 10–50. Internal consistency in the sample was (*α* = .84).

##### 2.4.1.1. Secondary outcome measures evaluated symptoms of

PTSD using the *Posttraumatic Stress Disorder Checklist for DSM-5* (PCL-5) (Weathers et al., 2013) which assessed re-experiencing, avoidance, negative alterations in cognition and mood, and hyper-arousal using 20 self-report items. Participants completed the PCL-5 with reference to the bereavement they had experienced. Internal consistency was good (*α* = .88).

Depression using *Patient Health Questionnaire (PHQ-9)* (Kroenke et al., 2001). The PHQ-9 is a self-administered nine-item questionnaire designed to assess general depression and distress, in accordance with the criteria for major depressive disorder as per the DSM-IV-TR (American Psychiatric Association, 2000). Internal consistency was good (*α* = .89).

Anxiety using the Generalized Anxiety Disorder-7 (GAD-7) (Spitzer et al., 2006). A seven-item measure assessing the frequency of anxiety symptoms over the past two weeks. Internal consistency was good (*α* = .83).

Self-reported self-compassion using the Self-Compassion Scale–Short Form (SCS-SF) (Raes et al., 2011) is a 12-item self-report questionnaire. Scores range from 12 to 60. Internal consistency was good (*α* = .84).

##### 2.4.1.2. Process measures – The Oxford Grief Scales

**Negative grief appraisals** *(OG-A)* (Smith, 2018)**.** This 35-item questionnaire measures participants’ agreement with various statements over the past month (1 – totally disagree to 7 – totally agree). The items cover five domains: (1) Loss of self and life, (2) Regret, (3) Catastrophic consequences of grief, (4) Loss of relationships and future, and (5) Fear of losing connection to the deceased. Internal consistency was excellent (*α* = .95).

**Loss-related memory characteristics** *(OG-M)(*Smith et al., 2022*).* Twenty-seven items assessed the frequency and qualities of unintentional loss-related memories, memory triggers and their effects, memory qualities, and the physical impact of loss-related memories. Participants rated these items on a 5-point scale (0 – not at all to 4 – very strongly) based on their experiences in the past month. Internal consistency was excellent (*ω* = .94). This scale was in development during 2016 and was only finalized in time for inclusion in the questionnaire battery for cohort 3 in 2016 (waitlist). This meant that the OG-M was not available for the 27 participants in cohorts 1 and 2 in 2016.

**Unhelpful coping strategies** *(OG-CS)(*Smith et al., 2024*)*. This 23-item questionnaire evaluates the frequency of a variety of maladaptive coping strategies after loss. The items cover four domains: (1) Avoidance, (2) Proximity seeking, (3) Loss rumination and (4) Injustice rumination. Internal consistency was excellent (*α* = .90).

**Social Disconnection** *(OG-SD)* (Smith, Wild, et al., 2020). This 15-item questionnaire assesses self perceived social disconnection after loss. Participants rate agreement (1 – totally disagree to 7 – totally agree) on 3 content domains (1) Negative interpretations of other people’s reactions to expressions of grief (2) a sense that an individual’s social self has been altered by the bereavement, (3) feeling a sense of safety in solitude. Internal consistency was excellent (*α* = .91).

#### 2.4.2. Data analysis

Descriptive statistics are reported for data regarding participant demographics. Change on the outcome and process variables over time was examined using linear mixed effect models, which have the advantage of being able to account for repeated measures, nested data structures and data missing at random. The primary analysis examined data from all participants who started treatment. Time (pretreatment, posttreatment, and 3-month follow-up) was specified as a categorical fixed factor, with a random effect of participants nested within cohorts, to account for variation between individuals. All models used restricted maximum likelihood estimation and an unstructured covariance matrix. Q-Q plots indicated that the normality of residuals assumption was met for all models. Within-group effect sizes were calculated from baseline using the baseline standard deviation. 95% confidence intervals for *d*_Cohen_ were calculated by dividing the upper and lower limits of the adjusted group difference by the baseline standard deviation.

We then performed a secondary analysis exploring whether participants allocated to the waitlist changed significantly during the waiting period on outcome measures (e.g. Time (pre-wait, pretreatment)). This analysis combined participants who completed a wait period which included those who were randomized and those allocated by availability. Otherwise, models were as described above.

The examination of possible predictors of outcome was performed using linear regression, with the posttreatment score on the adapted PG-13 as the dependent variable. Pretreatment PG-13-R scores were entered in the first step, then the variable of interest in step two. All analyses were performed in R version 4.0.3 (R Core Team, 2023) using the ‘tidyverse’ (Wickham et al., 2019), ‘nlme’ (Pinheiro et al., 2018), ‘jmv’ (Selker, Love, & Dropmann, 2018) and ‘psych’ (Revelle & Revelle, 2015) packages. All analyses were performed on the intention to treat sample using an alpha level of *p* = .05 with no imputation. Participants were considered missing if end-of-treatment or follow-up data could not be obtained.

## 3. Results

**Figure 1.**
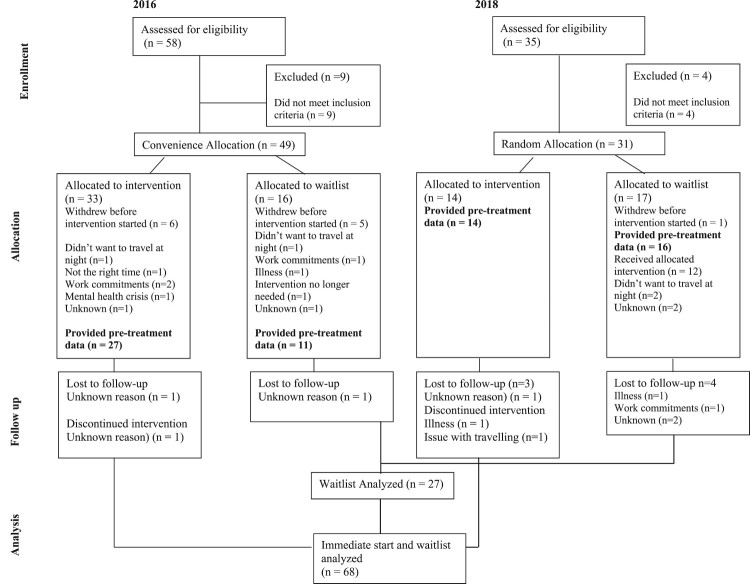
Enrolment and allocation.

A total of 80 adults were eligible for the study and were enrolled into a group. Of these, 33 were allocated to a wait period prior to starting treatment. Six participants in the immediate start and six in the waitlist groups did not provide pre-treatment data and were therefore excluded from the analysis. See Figure 1 for enrolment and allocation information. Descriptives of demographic variables for the 68 provided baseline data are shown in Table 1.

**Table 1.** Demographic details of the study sample.

Variable	Total Sample (*n* = 68)
Age: Mean (SD)	47.48 (15.09)
% Female	85
Ethnicity	
White or Caucasian	55 (81%)
Asian or Asian British: Indian	3 (4%)
Asian or Asian British: Pakistani	0
Asian or Asian British: Bangladeshi	0
Asian or Asian British: Chinese	2 (3%)
Asian or Asian British: Other Asian	1 (1%)
Black African or Black British	2 (3%)
Black Caribbean or Black British	0
Other	3 (4%)
Missing	2 (3%)
Education	
No qualifications	1 (1%)
High School level	15 (22%)
Undergraduate level	34 (50%)
Postgraduate level	16 (24%)
Missing	2 (3%)
Months since death: Mean (SD)	29.18 (34.10)
Who died	
Child	3 (4%)
Partner	27 (40%)
Parent	24 (35%)
Sibling	8 (12%)
Other relative	4 (6%)
Close non-relative	1 (1%)
Missing	1 (1%)

Note: % Figures rounded to nearest whole number.

Sixty-eight participants provided baseline data. Sixty five per cent (*N* = 44/68) met criteria for probable PGD with a score of 30 or above. Of those in the immediate start groups 83% (*N* = 34/41) provided end-of-treatment and follow-up data (see Figure 1). Of those in the waitlist group 63% (*N* = 17/27) provided end of treatment and follow-up data. Most participants (54.4%; *n* = 37) attended all group sessions, with 26% (*n* = 18) attending at least four, and 17.6% (*n* = 12) fewer than four but more than one.

**Figure 2.**
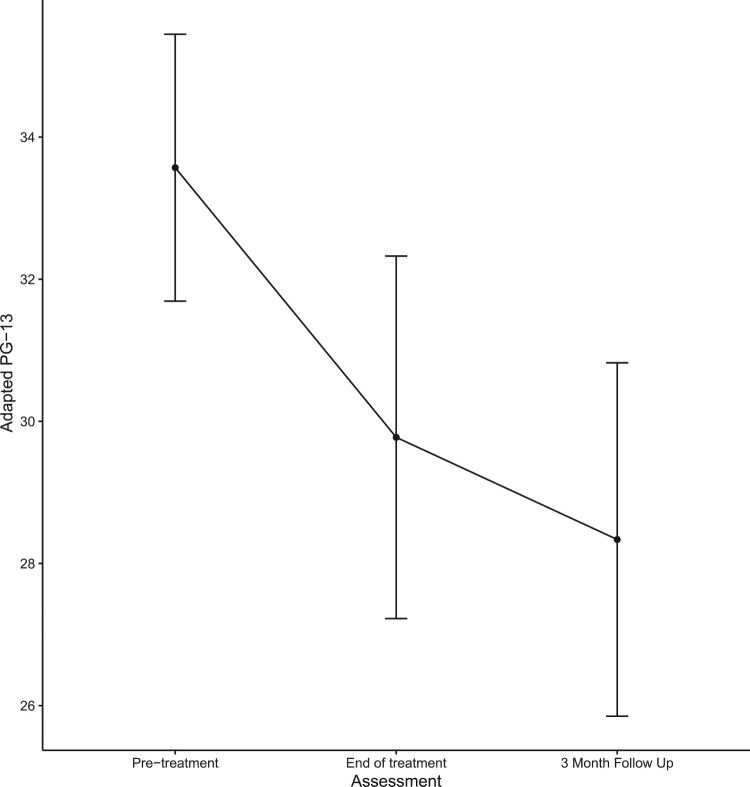
Mean scores on the PG-13 at each time point. Error bars = 95%CI.

Three of the 33 allocated to the waitlist group did not provide pre-wait data therefore changes in PG-13 scores during the waiting period were examined for the 30 participants. The linear mixed effect model showed that scores did not significantly change between baseline and the start of the intervention (estimate = −1.83, *SE* = 0.90, *p* > .05). Table 2 reports the unadjusted means and standard deviations, adjusted differences, and effect sizes for the outcome and process variables. Mean PG-13 scores at each time point are shown in Figure 2. Results suggest that the intervention was associated with significant reductions in symptoms of PGD, PTSD, Depression, and Anxiety, and a significant increase in self-compassion. The within-group *d*_Cohen_ for the PG-13 was 0.40 at posttreatment, and 0.65 at 3-month follow-up, indicating a small to medium-sized effect.

**Table 2.** Unadjusted means, adjusted differences, and effect sizes on the clinical outcome and process variables.

Measure	Time	Unadjusted Mean (SD) [*N*]	Adjusted difference [95%CI], *p* value	Within-Group Effect size *d*_Cohen_ [95%CI]
PG-13	Pre	33.57 (7.76) [68]		
	Post	29.78 (8.88) [49]	−3.09 [−4.59, −1.59], <.001	0.40 [0.21, 0.59]
	FU	28.34 (8.84) [51]	−5.02 [−6.49, −3.54], <.001	0.65 [0.46, 0.84]
PCL-5	Pre	33.54 (13.86) [68]		
	Post	28.44 (15.99) [49]	−4.06 [−6.86, −1.26], .005	0.29 [0.09, 0.50]
	FU	27.18 (15.93) [51]	−6.11 [−8.87, −3.36], <.001	0.44 [0.24, 0.64]
PHQ-9	Pre	10.40 (6.15) [68]		
	Post	8.78 (6.94) [49]	−1.34 [−2.58, −0.09], .035	0.22 [0.01, 0.42]
	FU	8.24 (5.95) [51]	−1.96 [−3.18, −0.73], .002	0.32 [0.12, 0.52]
GAD-7	Pre	9.41 (5.50) [68]		
	Post	7.76 (6.03) [49]	−1.51 [−2.87, −0.15], .030	0.27 [0.03, 0.52]
	FU	7.82 (6.04) [51]	−1.56 [−2.91, −0.22], .023	0.28 [0.04, 0.53]
SCS	Pre	30.43 (7.87) [68]		
	Post	34.88 (8.83) [49]	4.20 [2.36, 6.04], <.001	0.53 [0.30, 0.77]
	FU	34.10 (8.94) [50]	3.52 [1.70, 5.35], <.001	0.45 [0.22, 0.68]
OG-A	Pre	134.86 (40.49) [68]		
	Post	125.64 (47.01) [48]	−5.39 [−12.46, 1.69], .134	0.13 [−0.04, 0.31]
	FU	123.62 (43.57) [50]	−10.05 [−17.02, −3.09], .005	0.25 [0.08, 0.42]
OG-CS	Pre	59.79 (16.68) [68]		
	Post	56.90 (19.83) [48]	−1.10 [−4.02, 1.81], .453	0.07 [−0.11, 0.24]
	FU	55.60 (19.06) [50]	−3.44 [−6.31, −0.57], .019	0.21 [0.03, 0.38]
OG-M	Pre	56.02 (22.62) [41]		
	Post	48.12 (26.41) [26]	−6.89 [−12.3, −1.49], .013	0.30 [0.07, 0.54]
	FU	49.94 (27.03) [26]	−7.22 [−12.62, −1.82], .01	0.32 [0.08, 0.56]
OG-SD	Pre	67.56 (17.72) [68]		
	Post	68.90 (19.59) [48]	2.44 [−1.27, 6.16], .194	0.14 [−0.07, 0.35]
	FU	66.10 (18.81) [50]	−1.32 [−4.97, 2.34], .475	0.07 [−0.13, 0.28]

Notes: Pre = Pre-treatment, Post = Post-treatment, FU = 3-month Follow-up PG-13 = Prolonged Grief Disorder Inventory – 13 PCL-5 =  Posttraumatic Stress Disorder Checklist for DSM-5. PHQ-9 = Patient Health Questionnaire. GAD-7 = Generalized Anxiety Disorder-7, SCS = Self-compassion Scale Short Form. OG-A = The Oxford Grief Appraisals Scale, OG-CS = The Oxford Grief Coping Strategies Scale, OG-M = The Oxford Grief Memory Characteristics Scale, OG-SD = The Oxford Grief Social Disconnection Scale.

Results for the process measures are given in Table 2. These indicated a significant reduction in loss-related memory characteristics was observed at the posttreatment (*d* = .30) and follow-up timepoints (*d* = .32). Significant reductions in negative appraisals (*d* = .25) and unhelpful coping strategies (*d* = .21) were observed at follow-up, but not at post-treatment, suggesting that the changes on these variables occurred over a longer time period. Social disconnection did not show significant change at either the posttreatment or follow-up timepoints.

Lastly, exploratory linear regression analyses were used to examine possible predictors of clinical outcome (posttreatment scores on the PG-13-R). Results showed that the cognitive appraisals score at the start of the intervention was a significant predictor of improvements in PGD: standardized beta = 0.339, *p* = .004, indicating that a lower baseline appraisals score predicted a larger reduction in grief severity. No significant predictive effects were observed for the other process variables or demographic characteristics examined in the study (all *p* > .05).

## 4. Discussion

Consistent with previous research (Jerome et al., 2018) the results of this service evaluation demonstrated a positive and sustained therapeutic effect of the Loss Foundation’s group protocol for individuals bereaved by cancer. Participants who waited 3 months for treatment showed no significant changes in their grief, suggesting that the therapeutic effects are unlikely due to spontaneous recovery. However, due to limited numbers in the randomized cohorts, we are unable to make stronger conclusions about whether the group protocol performed significantly better than waitlist, and further randomized studies are needed.

All symptom measures showed significant reductions from baseline to the end of treatment and follow-up, with the strongest effects observed for PGD. Effect size estimates for the change in grief symptoms indicated a small effect at the end of treatment, and a medium effect at three-month follow-up. This is particularly encouraging given that participants were not selected based on grief severity. Approximately one third of participants started treatment below the clinical threshold for PGD, limiting the extent of measurable change over time. The findings suggest that those with milder symptoms at baseline can still benefit from the intervention, and are in line with other research demonstrating the effectiveness of psychotherapeutic group-based approaches in reducing PGD symptoms among those bereaved by cancer (Lacasta & Cruzado, 2024).

PTSD, depression and anxiety all showed significant reductions at the end of treatment that were sustained at follow-up. Although the effect sizes were small, this is consistent with the fact that baseline scores were not in the severe range, limiting the potential for larger improvements from therapeutic intervention. This outcome aligns with recent research indicating that mindfulness and self-compassion interventions can lead to significant reductions in depression and anxiety, even in populations with mild to moderate baseline symptoms (Han & Kim, 2023; Takahashi et al., 2019). For example, a meta-analysis has shown that self-compassion-based interventions are effective in alleviating depressive symptoms, anxiety, and stress across various settings, suggesting the robustness of these therapeutic approaches in enhancing emotional regulation (Han & Kim, 2023).

Moreover, the intervention significantly increased self-reported self-compassion by the end of treatment, with a moderate effect size (*d* = .53). Compassion-based approaches posit that the body’s emotion regulation systems – threat, drive, and soothing – are interconnected, with the activation of one system deactivating the others (Gilbert, 2016). In this study, participants reflected on whether their bereavement had led to a hyperactive threat system and were taught strategies to activate their self-soothing system. Techniques such as creating a ‘perfect nurturer’ – an imaginal entity capable of providing comfort and encouraging positive actions – were employed (Lee, 2005). These findings support previous research that links higher levels of self-compassion with less severe symptoms of prolonged grief disorder (PGD) (Lenferink et al., 2017), even though other studies have reported mixed results regarding the effectiveness of group-based Compassion Focused Therapy (CFT) in reducing PGD symptoms (Johannsen et al., 2022). These results suggest that self-compassion can be effectively developed in bereavement settings when delivered alongside CBT-based tools, potentially leading to broader improvements in mental health outcomes. The intervention’s impact on reducing PTSD, depression, and anxiety, alongside increasing self-compassion, underscores the value of incorporating compassion-based approaches in therapeutic settings, particularly for individuals dealing with grief and loss.

### 4.1. Process measures

Significant reductions in memory characteristics were observed by the end of treatment and at follow-up. This supports the idea that as the loss memory is activated, engaged with, and reprocessed the distressing nature, emotional salience, ease of activation and visceral consequences of the loss memories decrease. Therapeutic work within the groups involved psychoeducation of the nature of traumatically encoded memories and impact of avoidance on loss integration. Session 7 encouraged participants to identify the worst meaning of their difficult loss memories and shift their focus on to how they carried their loved one with them now (Wild et al., 2023). Due to data availability, we are unable to determine conclusively that changes in memory characteristics preceded grief symptom reductions, but this provides strong evidence for the interplay between these two processes.

Significant reductions in negative appraisals and maladaptive coping strategies were observed between baseline and follow-up, suggesting that these cognitive variables may require more time to be influenced by therapeutic intervention. The intervention described in this study is a brief, 7-week programme with a maximum of 14 h of facilitator time, making it resource-efficient for a group protocol treating up to 20 individuals. It is possible that these processes could be more effectively targeted individually through careful identification of specific appraisals or coping strategies. Alternatively, the group protocol may not have focused sufficiently on these factors to induce change.

Interestingly, social disconnection did not significantly improve at posttreatment or at follow-up. One might have expected that the experience of being in a group with other bereaved individuals would foster reciprocity and a shared understanding of loss. Indeed, participants frequently reported the significant benefits of being with others who ‘get it.’ However, despite this bonding and shared understanding, the effects may not have extended into their broader social circles, leaving participants still feeling disconnected in the social settings they frequented most.

Finally, results showed that negative appraisals at baseline were a significant predictor of treatment outcome with those reporting lower levels of grief-related appraisals being more likely to respond to treatment. This finding is in line with previous research that found lower levels of negative appraisals measured soon after a loss were linked with more adaptive grief trajectories in the first 12–18 months after a bereavement (Smith & Ehlers, 2020). Treatment studies have also shown that certain beliefs around the centrality of the loss and counterfactual thinking were associated with poorer treatment outcomes in a sample meeting criteria for complicated grief (Skritskaya et al., 2020). One explanation for this may be that due to the format of this brief group-based intervention idiosyncratic grief-related appraisals that may have benefited from individual cognitive restructuring were not fully explored. Future studies could helpfully investigate how best to achieve this within a group format.

These results build on previous findings from Jerome et al. (2018) by expanding the study to two additional cohorts, confirming significant reductions in symptoms of PGD, PTSD, anxiety, and depression, as well as improvements in self-compassion. This study also extends earlier work by documenting changes in cognitive–behavioural mechanisms relevant to the development and maintenance of PGD. However, several limitations should be noted.

First, the study design does not allow for definitive conclusions about the effectiveness of the group protocol in comparison to what might occur during a waiting period. As with Jerome et al. (2018) the grief severity of participants on the waitlist did not significantly change during the 3-month wait period. However, due to the lack of randomization across all cohorts, we cannot confidently determine whether the waitlist group differed from the immediate-start groups in ways that could have affected outcomes, nor can we attribute the observed clinical improvements solely to the intervention.

Limitations in resources during the recruitment period led to fewer groups being run in 2018 than originally planned, which resulted in underpowered randomized analyses. Combining data from the 2016 and 2018 cohorts for this service evaluation meant that group allocation methods and intervention delivery (6 versus 7 sessions) differed, potentially introducing variability in the outcomes and limiting the generalizability of the findings. Future research should replicate these results with randomized cohorts and larger sample sizes to ensure more robust conclusions and establish causality. While therapists worked through the manual in each session providing the same introductory content, techniques, and structure across all of the groups no formal empirical measure of treatment fidelity was recorded. Future research would benefit from the groups being recorded and assessed for adherence to protocol by an independent rater.

Another limitation concerns data completeness, as approximately 25% of participants did not provide end-of-treatment or follow-up data. This may have been due to limited resources for reminding participants to complete their questionnaires. However, attrition once the groups began was relatively low, with 72% attending more than half of the sessions, which can be considered a strength. Nevertheless, some participants dropped out before the intervention started, often due to practical difficulties in being able to attend in person. A clearer screening process could help reduce pre-intervention dropout in future studies. Additionally, the memory characteristics questionnaire was only completed by three cohorts, limiting the available data. Although the results showed significant findings, further studies are needed to determine its role as a potential mechanism of change in treating PGD. This paper did not examine the impact of cognitive–behavioural process measures on other outcomes, such as PTSD, depression, and anxiety. Prior research by (Smith & Ehlers, 2023) has identified memory characteristics, appraisals, and coping strategies as mechanisms in post-loss PTSD and depression. Future analyses could explore the influence of these factors on broader psychological treatment outcomes.

Finally, due to the real-world, pragmatic nature of this study within a working charitable organization, the primary and secondary outcomes relied on self-report measures. While self-reporting can sometimes overestimate the prevalence of mental health issues (Lim et al., 2018), future studies might consider using blind assessors and structured clinical interviews, as some have recently been validated for PGD (O’Connor et al., 2025; Rueger et al., 2024).

In conclusion, this service evaluation of The Loss Foundation’s therapeutic group intervention for individuals bereaved by cancer demonstrates promising outcomes, particularly in reducing symptoms of PGD, PTSD, depression, and anxiety, while enhancing self-compassion. These findings align with cognitive–behavioural models and suggest that targeted interventions addressing maladaptive thoughts, coping strategies, and loss-related memory characteristics can facilitate meaningful improvements in mental health following a loss. However, limitations such as the lack of randomization, incomplete data, and variability in group delivery highlight the need for further randomized controlled trials with larger sample sizes to validate these results and explore causal mechanisms. Despite these constraints, this study provides valuable insight into the potential effectiveness of group-based interventions for grief and lays a foundation for future research to refine therapeutic approaches and ensure broader applicability in real-world settings.

## Supplementary Material

Supplementary Material.docx

## Data Availability

No data arising from the 2016 groups can be shared publicly in line with the terms of the ethics agreement provided by University College London Research Ethics Committee (project ID: CEHP/2015/530). Data relating to the 2018 groups can be requested from the last author, ET. Readers can request analyses scripts and corresponding outputs from the first author KS.

## Note

1 See supplementary materials for more information.

## References

[CIT0001] American Psychiatric Association. (2000). *Diagnostic statistical manual of mental disorders* (revised 4th ed. text rev.).

[CIT0002] American Psychiatric Association. (2022). *Diagnostic and statistical manual of mental disorders* (5th ed., text rev.). 10.1176/appi.books.9780890425787.

[CIT0003] Boelen, P. A., van den Hout, M. A., & van den Bout, J. (2006). A cognitive-behavioral conceptualization of complicated grief. *Clinical Psychology: Science and Practice*, *13*(2), 109–128. 10.1111/j.1468-2850.2006.00013.x

[CIT0004] Bray, F. L. M., Sung, H., Ferlay, J., Siegel, R. L., Soerjomataram, I., & Jemal, A. (2024). Global cancer statistics 2022: GLOBOCAN estimates of incidence and mortality worldwide for 36 cancers in 185 countries. *CA: A Cancer Journal for Clinicians*, *74*(3), 229–263. 10.3322/caac.21834 38572751

[CIT0005] Ehlers, A., & Clark, D. M. (2000). A cognitive model of posttraumatic stress disorder. *Behaviour Research and Therapy*, *38*(4), 319–345. 10.1016/S0005-7967(99)00123-0 10761279

[CIT0006] Gilbert, P. (2009). Introducing compassion-focused therapy. *Advances in Psychiatric Treatment*, *15*(3), 199–208. 10.1192/apt.bp.107.005264

[CIT0007] Gilbert, P. (2016). *Human nature and suffering*. Routledge.

[CIT0008] Guldin, M.-B., Vedsted, P., Zachariae, R., Olesen, F., & Jensen, A. B. (2012). Complicated grief and need for professional support in family caregivers of cancer patients in palliative care: A longitudinal cohort study. *Supportive Care in Cancer*, *20*(8), 1679–1685. 10.1007/s00520-011-1260-3 21892795

[CIT0009] Han, A., & Kim, T. H. (2023). Effects of self-compassion interventions on reducing depressive symptoms, anxiety, and stress: A meta-analysis. *Mindfulness*, *14*(7), 1553–1581. 10.1007/s12671-023-02148-x PMC10239723 37362192

[CIT0010] Jerome, H., Smith, K. V., Shaw, E. J., Szydlowski, S., Barker, C., Pistrang, N., & Thompson, E. H. (2018). Effectiveness of a cancer bereavement therapeutic group. *Journal of Loss and Trauma*, *23*(7), 574–587. 10.1080/15325024.2018.1518772 PMC6461201 30983910

[CIT0011] Johannsen, M., Schlander, C., Farver-Vestergaard, I., Lundorff, M., Wellnitz, K. B., Komischke-Konnerup, K. B., & O'Connor, M. (2022). Group-based compassion-focused therapy for prolonged grief symptoms in adults – results from a randomized controlled trial. *Psychiatry Research*, *314*, Article 114683. 10.1016/j.psychres.2022.114683 35717855

[CIT0012] Killikelly, C., & Maercker, A. (2018). Prolonged grief disorder for ICD-11: The primacy of clinical utility and international applicability. *European Journal of Psychotraumatology*, *8*(Suppl 6), Article 1476441. 10.1080/20008198.2018.1476441 PMC5990943 29887976

[CIT0013] Kim, Y., Shaffer, K. M., Carver, C. S., & Cannady, R. S. (2014). Prevalence and predictors of depressive symptoms among cancer caregivers 5 years after the relative’s cancer diagnosis. *Journal of Consulting and Clinical Psychology*, *82*(1), 1–8. 10.1037/a0035116 24364792

[CIT0014] Kroenke, K., Spitzer, R., & Williams, J. (2001). The PHQ-9: Validity of a brief depression severity measure. *Journal of General Internal Medicine*, *16*(9), 606–613. 10.1046/j.1525-1497.2001.016009606.x 11556941 PMC1495268

[CIT0015] Lacasta, M. A., & Cruzado, J. A. (2024). Effectiveness of a cognitive–behavioral group therapy for complicated grief in relatives of patients with cancer: A randomized clinical trial. *Palliative and Supportive Care*, *22*(5), 954–960. 10.1017/S147895152300010X 36825484

[CIT0016] Lechner-Meichsner, F., Mauro, C., Skritskaya, N. A., & Shear, M. K. (2022). Change in avoidance and negative grief-related cognitions mediates treatment outcome in older adults with prolonged grief disorder. *Psychotherapy Research*, *32*(1), 78–90. 10.1080/10503307.2021.1909769 33818302 PMC8490492

[CIT0017] Lee, D. A. (2005). The perfect nurturer: A model to develop a compassionate mind within the context of cognitive therapy. In P. Gilbert (Ed.), *Compassion* (pp. 326–351). Routledge.

[CIT0018] Lenferink, L. I. M., Eisma, M. C., de Keijser, J., & Boelen, P. A. (2017). Grief rumination mediates the association between self-compassion and psychopathology in relatives of missing persons. *European Journal of Psychotraumatology*, *8*(sup6), Article 1378052. 10.1080/20008198.2017.1378052 PMC5687807 29163871

[CIT0019] Lim, G. Y., Tam, W. W., Lu, Y., Ho, C. S., Zhang, M. W., & Ho, R. C. (2018). Prevalence of depression in the community from 30 countries between 1994 and 2014. *Scientific Reports*, *8*(1), 2861. 10.1038/s41598-018-21243-x 29434331 PMC5809481

[CIT0020] Näppä, U., & Björkman-Randström, K. (2020). Experiences of participation in bereavement groups from significant others’ perspectives; a qualitative study. *BMC Palliative Care*, *19*(1), 124. 10.1186/s12904-020-00632-y 32799845 PMC7429679

[CIT0021] O’Connor, M., Vang, M. L., Bryant, R. A., Buur, C., Komischke-Konnerup, K. B., Frostholm, L., & Ladegaard, N. (2025). Development and validation of the Aarhus Structured Clinical Interview for Prolonged Grief Disorder in ICD-11 and DSM-5-TR (A-PGDi). *European Journal of Psychotraumatology*, *16*(1), Article 2511373. 10.1080/20008066.2025.2511373 PMC12180314 40534423

[CIT0022] Pinheiro, J., Bates, D., DebRoy, S., & Sarkar, D. (2018). R-Core-Team. (2014). *Nlme: Linear and nonlinear mixed effects models*, 3.1-118.

[CIT0023] Prigerson, H. G., Boelen, P. A., Xu, J., Smith, K. V., & Maciejewski, P. K. (2021). Validation of the new DSM-5-TR criteria for prolonged grief disorder and the PG-13-Revised (PG-13-R) scale. *World Psychiatry*, *20*(1), 96–106. 10.1002/wps.20823 33432758 PMC7801836

[CIT0024] Prigerson, H. G., & Maciejewski, P. K. (2008). *Prolonged grief disorder (PG-13) scale*. Dana-Farber Cancer Institute.

[CIT0025] Raes, F., Pommier, E., Neff, K. D., & Van Gucht, D. (2011). Construction and factorial validation of a short form of the self-compassion scale. *Clinical Psychology & Psychotherapy*, *18*(3), 250–255. 10.1002/cpp.702 21584907

[CIT0025a] R Core Team. (2023). R: A language and environment for statistical computing. [Computer software]. Retrieved from https://www.R-project.org/

[CIT0026] Revelle, W., & Revelle, M. W. (2015). Package ‘psych’. *The Comprehensive R Archive Network*, *337*(338), 161–165.

[CIT0027] Rodriguez-Villar, S., Okegbola, E. O., Arevalo-Serrano, J., Duval, Y., Mathew, A., Rodriguez-Villar, C., Smith, K. V., Kennedy, R. C., & Prigerson, H. G. (2024). Grief and coping among relatives of patients who died of COVID-19 in intensive care during the height of the COVID-19 pandemic. *BJPsych Open*, *10*(6), e181. 10.1192/bjo.2024.741 39402964 PMC11698220

[CIT0028] Rueger, M. S., Lechner-Meichsner, F., Kirschbaum, L., Lubik, S., Roll, S. C., & Steil, R. (2024). Prolonged grief disorder in an inpatient psychiatric sample: Psychometric properties of a new clinical interview and preliminary prevalence. *BMC Psychiatry*, *24*(1), 333. 10.1186/s12888-024-05784-2 38693470 PMC11064282

[CIT0028a] Selker, R., Love, J., & Dropmann, D. (2018). jmv: The “jamovi” analyses (R Package Version 0. 8.1.14). Retrieved from https://CRAN.R-project .org/packagejmv

[CIT0029] Skritskaya, N. A., Mauro, C., Garcia de la Garza, A., Meichsner, F., Lebowitz, B., Reynolds, C. F., Simon, N. M., Zisook, S., & Shear, M. K. (2020). Changes in typical beliefs in response to complicated grief treatment. *Depression and Anxiety*, *37*(1), 81–89. 10.1002/da.22981 31804005 PMC6952544

[CIT0030] Smith, K. V. (2018). *Memories, appraisals, and coping strategies in prolonged grief disorder*. University of Oxford.

[CIT0031] Smith, K. V., & Ehlers, A. (2020). Cognitive predictors of grief trajectories in the first months of loss: A latent growth mixture model. *Journal of Consulting and Clinical Psychology*, *88*(2), 93–105. 10.1037/ccp0000438 31556649 PMC6939605

[CIT0032] Smith, K. V., & Ehlers, A. (2021). Prolonged grief and posttraumatic stress disorder following the loss of a significant other: An investigation of cognitive and behavioural differences. *PLoS One*, *16*(4), e0248852. 10.1371/journal.pone.0248852 33793567 PMC8016232

[CIT0033] Smith, K. V., & Ehlers, A. (2023). Coping strategies as a mediator of the effect of loss-related memory characteristics and negative loss-related appraisals on symptoms of PGD, PTSD and depression. *Psychological Medicine*, *53*(4), 1542–1551. 10.1017/S0033291721003123 37010218 PMC10009377

[CIT0034] Smith, K. V., Rankin, H., & Ehlers, A. (2020). A qualitative analysis of loss-related memories after cancer loss: A comparison of bereaved people with and without prolonged grief disorder. *European Journal of Psychotraumatology*, *11*(1), Article 1789325. 10.1080/20008198.2020.1789325 PMC7534291 33062204

[CIT0035] Smith, K. V., Thew, G. R., & Graham, B. (2018). Conducting ethical internet-based research with vulnerable populations: A qualitative study of bereaved participants’ experiences of online questionnaires. *European Journal of Psychotraumatology*, *9*(sup1), Article 1506231. 10.1080/20008198.2018.1506231 PMC6104613 30151076

[CIT0036] Smith, K. V., Wild, J., & Ehlers, A. (2020). The masking of mourning: Social disconnection and its relationship to psychological distress after loss. *Clinical Psychological Science*, *8*(3), 464–476. 10.1177/2167702620902748 32550046 PMC7252572

[CIT0037] Smith, K. V., Wild, J., & Ehlers, A. (2022). Psychometric characteristics of the Oxford Grief Memory Characteristics Scale and its relationship with symptoms of ICD-11 and DSM-5-TR prolonged grief disorder. *Frontiers in Psychiatry*, *13*. 10.3389/fpsyt.2022.814171 PMC8970310 35370837

[CIT0038] Smith, K. V., Wild, J., & Ehlers, A. (2024). From loss to disorder: The influence of maladaptive coping on prolonged grief. *Psychiatry Research*, *339*, Article 116060. 10.1016/j.psychres.2024.116060 PMC11513616 39068899

[CIT0039] Spitzer, R. L., Kroenke, K., Williams, J. B., & Löwe, B. (2006). A brief measure for assessing generalized anxiety disorder: The GAD-7. *Archives of Internal Medicine*, *166*(10), 1092–1097. 10.1001/archinte.166.10.1092 16717171

[CIT0040] Stroebe, M. S., & Schut, H. (1999). The dual process model of coping with bereavement: Rationale and description. *Death Studies*, *23*(3), 197–224. 10.1080/074811899201046 10848151

[CIT0041] Takahashi, T., Sugiyama, F., Kikai, T., Kawashima, I., Guan, S., Oguchi, M., Uchida, T., & Kumano, H. (2019). Changes in depression and anxiety through mindfulness group therapy in Japan: The role of mindfulness and self-compassion as possible mediators. *BioPsychoSocial Medicine*, *13*(1), 4. 10.1186/s13030-019-0145-4 30820241 PMC6378713

[CIT0042] Urbaniak, G. C., & Plous, S. (2013). *Research randomizer* (Version 4.0) [Computer software]*.* Retrieved June 22, 2013, from http://www.randomizer.org/

[CIT0043] Wanza, C., Gonschor, J., Smith, K. V., Ehlers, A., Barke, A., Rief, W., & Doering, B. K. (2023). Feeling alone in one's grief: Investigating social cognitions in adaption to bereavement using the German version of the Oxford Grief-Social Disconnection Scale. *European Journal of Trauma & Dissociation*, *7*(2), Article 100327. 10.1016/j.ejtd.2023.100327

[CIT0044] Weathers, F., Litz, B., Keane, T., Palmieri, P., Marx, B., & Schnurr, P. (2013). The PTSD checklist for DSM-5 (PCL-5). Scale available from the National Center for PTSD at www.ptsd.va.gov

[CIT0045] Wickham, H., Averick, M., Bryan, J., Chang, W., McGowan, L. D. A., François, R., Grolemund, G., Hayes, A., Henry, L., & Hester, J. (2019). Welcome to the Tidyverse. *Journal of Open Source Software*, *4*(43), 1686. 10.21105/joss.01686

[CIT0046] Wild, J., Duffy, M., & Ehlers, A. (2023). Moving forward with the loss of a loved one: Treating PTSD following traumatic bereavement with cognitive therapy. *The Cognitive Behaviour Therapist*, *16*, e12. 10.1017/S1754470X23000041 37159811 PMC10160000

