# Supplementary material for: Supporting those bereaved by cancer: a service evaluation and investigation of cognitive behavioural mechanisms in the treatment of prolonged grief
Source: Eur J Psychotraumatol. 2025 Sep 2;16(1):2545144. doi: 10.1080/20008066.2025.2545144 (PMC12406324; doi:10.1080/20008066.2025.2545144)
Supplement: Supplementary Material.docx [file ZEPT_A_2545144_SM6306.docx]

Supplementary Material

This material is copyrighted. To reproduce or request copies please email info@thelossfoundation.org.

The Loss Foundation Seven session therapeutic manual summary^©^

**Session 1 – Introductions & Orientation**

Session 1 lays the groundwork for the seven-week programme. The two psychologists welcome participants, clarify their roles, and underscore that the aim is not to erase grief but to develop healthier ways of living alongside it. Practicalities follow: the group meets evenings; brief online questionnaires before each session help the charity evaluate and refine the intervention.

Together the group co-creates ground rules—confidentiality, one voice at a time, phone-free space, no “shoulds,” and respect for diverse losses—so everyone feels safe. Participants then introduce themselves informally, sharing as much or as little about their bereavement as feels comfortable, and are invited to notice how speaking and listening affect them.

A flip-chart exercise explores “What grief looks like right now,” capturing physical, emotional and social effects, which validates the breadth of experiences in the room. A second chart asks “What do you hope to gain?”, steering expectations toward realistic goals such as coping strategies, peer connection and reduced distress rather than a promise to eliminate grief.

Facilitators teach and practise belly-breathing—explaining the physiological logic of calming the threat system—so everyone leaves with a first practical tool. Two homework tasks are set: practise this breathing daily and observe one current difficulty (e.g., poor sleep), noting typical responses. Journals are provided for these reflections, alongside an out-of-hours support sheet.

Throughout, the facilitators stress that all emotions are welcome, that the group is not a crisis service but thoughts of hopelessness can be openly discussed, and that courage includes simply turning up. By session’s end, members know the structure and ethos of the programme, feel initial connection with peers, and have begun building their coping toolkit.

**Session 2 – Grief, Anxiety, Low Mood & Mindfulness**

After revisiting key points and homework from Week 1, Session 2 dives into psycho-education. Facilitators outline three grief trajectories—acute, integrated and prolonged—and critically compare Kubler-Ross’s stages, the Dual Processing Model, and Tonkin’s “Growing Around Grief.” This normalises fluctuation and reinforces that grief need not shrink for life to expand.

The discussion shifts to anxiety after loss. Using a body outline, the group maps physiological signs (racing heart, dizziness) and learns why the fight-or-flight system misfires when the future feels uncertain. Low mood is then framed through a CBT “hot-cross-bun” diagram linking thoughts, feelings, sensations and behaviour; participants identify common ruminations (“Why didn’t I do more?”) and see how these drive withdrawal.

Attention turns to the power of thoughts: believing thoughts as facts can intensify distress, while observing them as mental events creates choice. Facilitators introduce mindfulness as present-moment, non-judgemental awareness that interrupts automatic spirals and increases flexibility.

Everyone practises “Mindfulness of the Hand,” a short sensory exercise that anchors attention in touch and movement. Homework includes: (1) daily use of the recorded mindfulness track, (2) noting three occasions when a distressing thought arose and its impact, and (3) capturing insights or questions for the next meeting. Hand-outs on anxiety, low mood and the Dual Processing Model support at-home review.

By the end of Session 2, participants share a richer language for grief’s forms, understand how anxiety and depression manifest in body and mind, and possess their first experiential mindfulness skill—setting the stage for applying self-care, compassion and cognitive tools in subsequent sessions.

**Session 3 – Flashbacks, Sleep & Everyday Self-Care**

Session 3 integrates trauma education with practical coping strategies. After homework reflections, facilitators demystify flashbacks, explaining them as sensory “snapshots” stored without time-stamp because high arousal hinders memory filing. The camera-and-album metaphor by Dr Kirsten Smith shows why intrusive images resurface and how avoidance keeps them raw.

A guided “safe-place” imagery exercise equips participants with a sensory grounding technique to counter flashback activation. Attention then turns to pervasive sleep problems. Common post-loss complaints (difficulty dropping off, early waking, nightmares) are normalised, and evidence-based tips follow: a consistent pre-bed wind-down, the “quarter of an hour rule”, reserving the bed for sleep, and a simple cognitive trick—silently repeating the word “the”—to occupy over-busy minds.

Broader self-care is framed as essential nervous-system maintenance. Facilitators highlight mood-enhancing nutrition, gentle exercise, fresh air and social contact, even if initially grief-specific, as small, predictable acts that rebuild safety. Micro-routines such as fixed mealtimes or a daily 11 am walk help re-establish physiological rhythm, which in turn supports emotional processing.

Hand-outs on flashbacks, sleep hygiene and self-care accompany three homework tasks: practise the imagery or breathing audio daily, identify one nourishing behaviour to repeat each day, and experiment with allowing (rather than pushing away) unwanted thoughts. The session closes with “Leaves on a Stream,” reinforcing acceptance-based mindfulness.

By week’s end, participants possess a cognitive model for flashbacks, a sensory grounding practice, concrete sleep-improvement steps, and a personalised routine framework—strengthening emotional regulation ahead of work on self-compassion and thinking styles.

**Session 4 – Cultivating Self-Compassion**

Building on previous coping skills, Session 4 introduces self-compassion as an antidote to the self-criticism that often intensifies grief. The group defines compassion, recognising suffering, feeling moved, responding with warmth, and distinguishes it from empathy or altruism. Three “flows” are explored: giving to others, receiving from others, and directing compassion inward. Most discover self-compassion is the toughest flow, especially after loss, when blame and “not doing enough” narratives loom large.

Facilitators outline the core components: kindness (versus judgment), common humanity (suffering links us), and mindfulness (balanced awareness of thoughts and feelings). Paul Gilbert’s model of emotion systems illustrates how grief over-activates threat and suppresses soothe; deliberate self-kindness re-engages the soothing system, releasing oxytocin and fostering resilience.

The experiential heart of the session is “Perfect Nurturer” imagery by Dr Deborah Lee. Participants imagine an entirely safe, benevolent figure—real, fictional or symbolic—who embodies unconditional acceptance. Guided visualisation helps them absorb this nurturing tone, which can later be invoked when self-criticism or shame flares.

Homework involves listening daily to the recorded compassion meditation, refine the nurturer image via a worksheet, and note three examples of using a kinder voice in everyday situations. An optional compassionate-letter template is supplied.

The session closes with a brief compassion meditation to anchor the experience somatically, and facilitators signpost ongoing supports. By the end, participants understand why self-compassion is critical post-bereavement, recognise personal barriers, and leave with both a conceptual map and an embodied practice to counter harsh self-talk.

**Session 5 – Responding to Unhelpful Thoughts**

Session 5 tackles the distressing cognitions that often accompany grief. Facilitators normalise “private grief thoughts” that feel frightening, shameful or endless, and outline how avoidance can shrink a person’s life. The “bungee run” analogy derived by Drs Erin Thompson and Kirsten Smith vividly demonstrates that sprinting away from pain snaps us back with equal force, reducing emotional range and blocking one end of the emotional spectrum (e.g., sadness) can dull access to its positive counterpart (e.g., joy).

Participants identify five common unhelpful thinking styles—critical self-talk, “shoulds,” mind-reading, catastrophising and black-and-white thinking—and learn to link each style to a matching response strategy:

1. Self-compassion softens harsh internal criticism.
2. Mindful distancing observes untestable worries (“I’ll never cope”) as mental events rather than facts.
3. Behavioural experiments test predictions that can be checked in real life (“If I cry, everyone will leave”).

A flip-chart matrix maps style → strategy, and a live case example shows belief ratings dropping after an experiment. Participants then practise classifying their own thoughts and brainstorm experiments under facilitator guidance.

Homework is a thought diary: record difficult thoughts, label the style, choose a response and, where possible, draft an experiment. Worksheets and the “bungee run” visual support at-home practice. The session ends with a mindful-eating exercise using raisins or sweets to reinforce present-moment attention.

By the close of Week 5 participants grasp why distressing cognitions are normal yet potent, understand how avoidance sustains them, and possess three concrete tools—compassion, mindfulness and hypothesis-testing—to loosen their grip and restore behavioural freedom.

**Session 6 – Behavioural Experiments & Transforming Flashbacks**

Session 6 deepens cognitive work by focusing on exposure through behavioural experiments and re-authoring distressing memories. The rationale is revisited: coping means altering one’s relationship with pain, not erasing it. Facilitators model a graded experiment—sharing feelings with a friend—and show how belief in the feared outcome (losing control) plummeted.

Participants design their own experiments using a worksheet, selecting predictions that feel challenging yet manageable and planning safety-net supports. Curiosity, not perfection, is emphasised.

Next, a guided imagery invites members to recall qualities their loved one fostered in them, kindness, humour, grit, and to sense these attributes permeating the body. The exercise culminates in choosing a symbolic image (e.g., a ray of sunshine) that represents the loved one’s ongoing influence.

This image is then woven into flashback work. Instead of disputing whether a flashback is painful, participants redirect meaning: juxtaposing the intrusive scene with the qualities that live on in them reframes the memory from “only loss” to “loss and legacy.” A table helps pair each flashback, its current meaning, the chosen qualities and a transformed meaning.

Homework includes completing the behavioural-experiment plan, updating the flashback-meaning table, and continuing mindfulness audio practice. Hand-outs capture key steps and provide examples.

The session closes with an instrumental mindful-listening exercise—attending to instruments’ entrances and exits—to reinforce focused attention. By the end, participants can design and evaluate experiments that disconfirm catastrophic beliefs, hold a personalised legacy image, and apply that image to soften intrusive memories—strengthening resilience ahead of the programme’s conclusion.

**Session 7 – Living by Values & Ending Well**

The final session integrates previous learning with a future-focused values framework. After a summary of the six prior meetings, members share a photo of their loved one and name qualities that person nurtured in them, reinforcing ongoing bonds.

Facilitators explain that values differ from goals: goals are endpoints, values are enduring directions that guide choices. Reconnecting with values broadens life around grief, echoing Tonkin’s model. Participants complete the “nine-moments” exercise—listing nine meaningful activities, then repeatedly crossing some off until three remain—to clarify what truly matters and contact the pain of potential losses.

Using an Acceptance and Commitment Therapy values bullseye, each person plots how closely current behaviour aligns with those top three values and identifies small, doable steps for moving inward (e.g., weekly coffee with friends to honour connection). The process highlights that grief can coexist with, and even deepen, purposeful living.

Group reflection invites participants to name their most helpful tools—breathing, safe-place imagery, self-compassion, experiments—and anticipate obstacles to sustaining change. Facilitators stress that awareness itself is therapeutic; not every technique will resonate immediately.

Practical endings follow: completion of post-programme questionnaires, information on follow-up surveys, and sign-posting to the charity’s peer groups, social events and external helplines. The meeting closes with a short mindful-movement sequence—stretching and body-gratitude—to anchor self-care physically.

By session’s end, participants have integrated cognitive-emotional skills with a values compass, honoured their loved ones’ legacies, and left with concrete next steps and support pathways, enabling them to continue living meaningfully while carrying their grief forward.
